# Abundance of Live and Dead Bacteriopsammon Inhabiting Sandy Ecosystems of Recreational Marine Beaches of the Southern Baltic Sea

**DOI:** 10.1007/s00248-022-02079-5

**Published:** 2022-07-25

**Authors:** Piotr Perliński, Zbigniew Jan Mudryk, Marta Zdanowicz, Łukasz Kubera

**Affiliations:** 1 Department of Experimental Biology, Institute of Biology and Earth Sciences, Pomeranian University in Słupsk, Arciszewskiego 22B str, 76-200 Słupsk, Poland; 2 Department of Microbiology and Immunobiology, Faculty of Biological Sciences, Kazimierz Wielki University, Al. Powstańców Wielkopolskich 10, 85-090 Bydgoszcz, Poland

**Keywords:** Abundance, Anthropopression, Bacteriopsammon, Baltic Sea, Beach, Live/dead, Sand

## Abstract

The study was carried out on four non-tidal sandy marine beaches located on the Polish part of the southern Baltic Sea coast. We applied a LIVE/DEAD™ *Bac*Light™ Bacterial Viability Kit (Invitrogen™) method to determine the abundance of live and dead bacteriopsammon. Live psammon bacteria cells constituted 31–53% of the total number of bacteria inhabiting sand of the studied beaches. Abundance of live and dead psammon bacteria generally differed along the horizontal profile in all beaches. The maximum density of bacteria was noted in the dune and the middle part of the beach (dry zones) and the minimum in wet zones, i.e., under seawater surface and at the swash zone. Generally along the vertical profile, the highest numbers of two studied bacterial groups were noted in the surface sand layer, while with increasing sediment depth their numbers significantly decreased. The abundance of live and dead bacteria showed a distinct seasonal variation.

## Introduction

Marine beaches present in all coasts constitute buffer zones between the atmosphere, land, and sea [1–3]. These marine coastal areas represent one of the largest ecosystems of the Earth and cover about 75% of the world’s unfrozen shorelines [4, 5]. Sandy beaches are characterised by the uncontrollable dynamic nature and their structure is determined by wind, sand, rainfall, variation in the rates of sun exposure, sea waves’ action, and water in the state of constant motion, therefore they are morphodynamically and climatically strongly differentiated [6, 7]. Marine beaches and coastal areas are also very often subject to considerable anthropogenic pressure due to their recreational function and holiday activities of millions of tourists mainly in the summer season [8, 9]. According to Velonakis et al. [2], time spent by sunbathers on the beaches is usually longer than that spent in seawater.

The permeable nature of sandy beach sediments allows for large quantities of seawater to pass through them over relatively short time. According to Brown and McLachlan [10] and Urban-Malinga et al. [9] sandy beaches can be regarded as gigantic filters through which large amounts of water are being filtered. Depending on whether a beach is dissipative or reflective between 1 and 100 m^3^ of seawater × m^2^ × v day can be flushed through the intertidal seawater via wave and tidal pumping and wave ramp [11, 12]. While seawater is being filtered, great amounts of organic compounds, mainly lipids, proteins, and carbohydrates from terrestrial, atmosphere, and marine environments are accumulated. These organic compounds are adsorbed onto the sand grain surface in the form of particulate organic matter (POM), and dissolved (DOM) organic matter [3, 9, 13]. POM and DOM are mineralized as seawater passes through sand, thus beaches play an important role in biogeochemical cycles of different nutrients, and also purification of these marine ecosystems [1, 14, 15].

Marine sandy ecosystems are inhabited by benthic prokaryote microbial community (micropsammon) and psammon bacteria constitute up to 90–99% of benthic biomass [16–18]. Psammon bacteria residing in interstidial spaces between sand grains live attached to surface grains as biofilm and play a key role in decomposition, biodegradation, transformation, and mineralization of organic matter accumulated during water filtration [1, 15]. According to Podgórska and Mudryk [19] and Astel et al. [3], about 70% of organic matter reaching the beach is being mineralized by metabolic and biochemical processes carried out by psammon bacteria. For this reason, these organisms function as an enormous biological filter, which plays a very important role in cleaning of such ecosystems [5, 9, 20]. Bacteria inhabiting the sand of the sea beach, which represents the border zone between land and sea, are colonized simultaneously by bacteria of terrestrial origin (limnotolerant) as well as bacteria of marine environment (halotolerant) [21]. Bacteriopsammon is characterized by high diversity. The physiological groups of bacteria colonizing the sand grains of sea beaches of the southern Baltic Sea are mainly dominated by ammonifying bacteria and uric acid hydrolyzing bacteria and the least numerous physiological group among the bacteriopsammon are sulphate-reducing bacteria [22]. Among the taxonomic composition of bacteria colonizing the sand of sea beaches in Czołpino and Sopot (southern Baltic Sea) bacteria of the genera *Acinetobacter* and *Microccocus* where predominant. Bacteria of the genera *Escherichia*, *Vibrio*, and *Photobacterium* were much less numerous [23].

The evaluation of the total bacterial number and their metabolic activity is thus essential in our understanding of their ecological role and their contribution to marine processes [24, 25]. Traditional direct counting technique of the total bacterial number applied by hydromicrobiologists using acridine orange and 4′,6-diamidino-2-phenylindole stains results in an overestimation as it includes also dead cells and consequently does not determine metabolically active and inactive fraction in aquatic ecosystems [26, 27]. For this reason, in recent years many different fluorescent staining techniques (6-carboxyfluorescein diacetate, 5-cyano-2, 3-ditolyl tetrazolium chloride, iodonitrotetrazolium chloride, fluorescein diacetate, SYBR® Green I and II) have been proposed to determine physiological state and metabolic activity of natural bacterial communities [25, 28]. One of the most commonly used method applied to determine metabolic activity of natural bacterial assemblages is commercially available LIVE/DEAD BacLight staining method [29, 30]. It allows discrimination between metabolic active (live) and metabolic inactive (dead) cells and is based on the cell membrane integrity and identification of a visible nucleoid region inside the bacterial cell [31, 32]. The LIVE/DEAD BacLight method is widely accepted as a rapid, simple, relatively precise counting and ecologically valuable technique of a quantitative measure of live and dead individual bacterial cells and which also provides a total count of bacteria [31, 33].

To our best knowledge, the current literature provides no example of the studies on the abundance of live and dead bacteria inhabiting sand of marine beaches. Therefore, with the LIVE/DEAD BacLight method, we aimed to provide reliable information on the seasonal number of live and dead bacteria and their spatial distribution in the sand of four beaches located in the Polish part of the southern Baltic Sea.

## Material and Methods

### Study Area

**Fig. 1:**
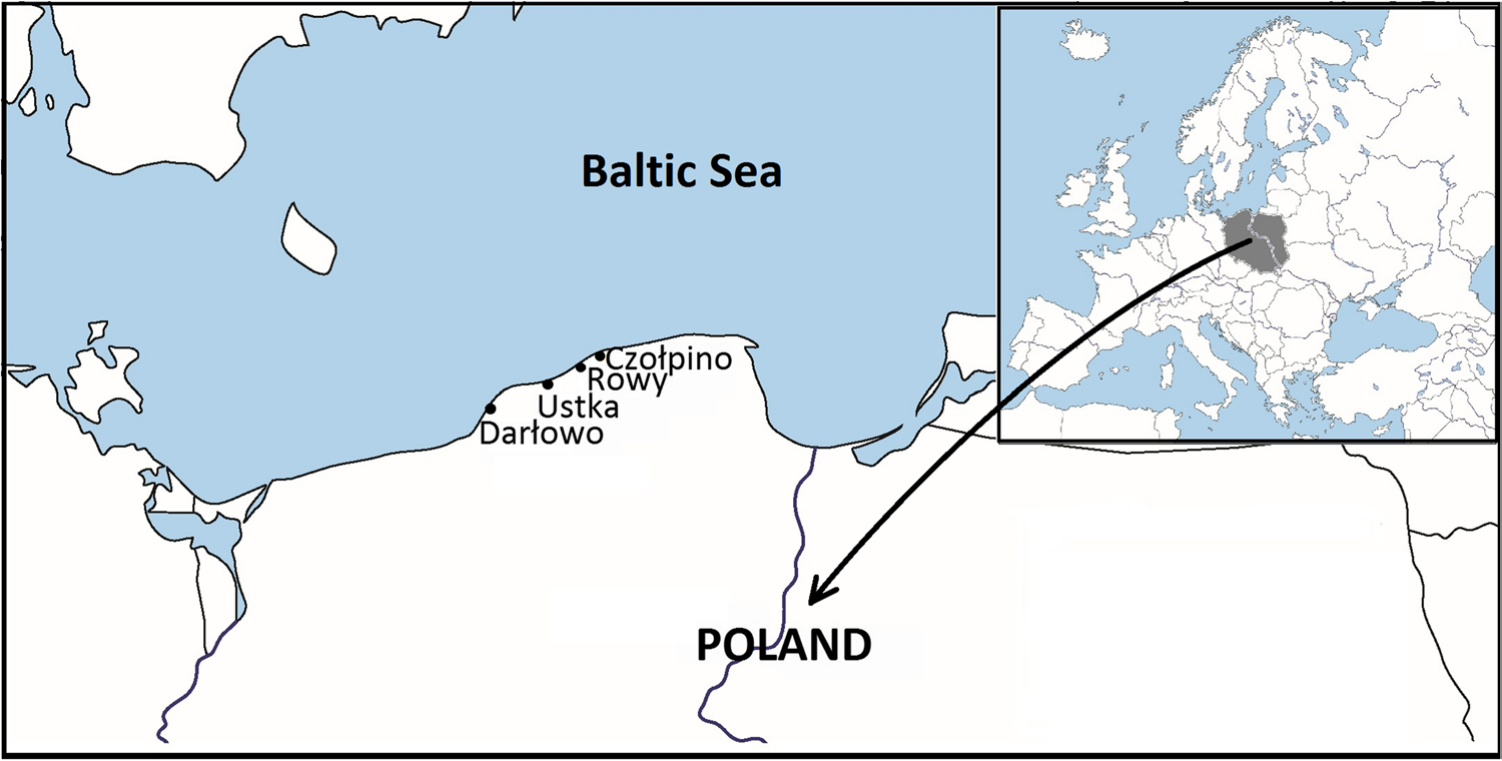
Location of the study beches (Czołpino, Darłowo, Rowy, Ustka) on north Poland coast of the Baltic Sea (https://d-maps.com/)

The study was carried out on four non-tidal sandy marine beaches located in Czołpino (54°43′N, 17°14′E), Darłowo (54° 25′N, 16° 24′E), Rowy (54°40′N, 17°3′E), and Ustka (54° 35′N, 16° 51′E), (southern Baltic Sea) (Fig. 1) and represent a dissipative beach type with longshore bars and troughs. They are classified as exposed and have an approximately slope of 7–9° while the width of the beach is about 45–85 m [20, 34]. In general, quartz sand of exposed beaches is fine and medium-grained, and about 90% of the sand grain size is between 0.125 and 0.250 mm [20]. The studied beaches, particularly in autumn and winter, are exposed to strong winds that generate high waves, which cause strong erosion onshore. As a result, the seashore along the studied beaches is heavily destroyed and the coastline retreats on average 0.10 m each year [35].

The studied beaches differ in their exploitation by recreation function and thus the level of anthropogenic pressure [3]. Moreover, the strength of anthropogenic impact on the studied beaches decreases in the following order: Ustka (very strong) > Darłowo (strong) > Rowy (moderate) > Czołpino (low). The Ustka and Darłowo beaches with their surf zone are one of the most picturesque, very popular bathing beaches and recreational areas in Poland. Polish and foreign tourists as well as local inhabitants intensively visit (about 40 to 500 thousand annually) these beaches, and they are usually very crowded mainly during the summer months. The study of Bigus et al. [36] also indicates that the analyzed beaches differing significantly in the influence of anthropopression are characterized by different levels of chemical parameters. The beach in Ustka had higher values of organic matter (4.53–34.45 mg × g^−1^) and organic carbon (93.06–20.46 mg × g^−1^) than the beach in Czołpino (organic matter 3.33–21.06 mg × g^−1^, organic carbon 0.92–19.68 mg × g^−1^). Also, the concentrations of heavy metals on the studied beaches indicate clear differences in the influence of anthropopressure on sandy beach sediments of a highly urbanized beach Ustka (Pb — 49.05 mg/kg, Mo — 70.95 mg/kg, Al — 106.87 mg/kg) and a beach Czołpino in a area under legal protection (Pb — 1.67 mg/kg, Mo — 25.89 mg/kg, Al — 76.30 mg/kg) [20]. The beach in Ustka is a municipal beach situated in the vicinity of a sea port and covers the area of 0.3 km^2^; it is dominated by fishing and tourist cruises. The beach in Rowy is used for recreational purposes to a much lesser extent than the beaches in Ustka and Darłowo. It is located in a very small resort and is one of the most popular nudist beaches in Poland, which significantly limits its general availability for recreational purposes. The beach in Czołpino is located in the Słowiński National Park, which is registered in the World Network of Biosphere Reserves. Characteristic protected elements within the Park are sandbars with moving dunes — unique in Europe. Dunes moving with the speed of 5–30 m per year are covered by saltgrass species such as: *Cakile maritima* ssp. *baltica*, *Honckenya peploides*, *Calammophila baltica* and *Linaria odora.* Due to the location in the national park, this beach is very rarely visited by tourists. This region is one of the least polluted in the Polish coastal zone, and as such, it meets one of the selection criteria of marine protected areas [20].

### Sampling Procedure

**Fig. 2:**
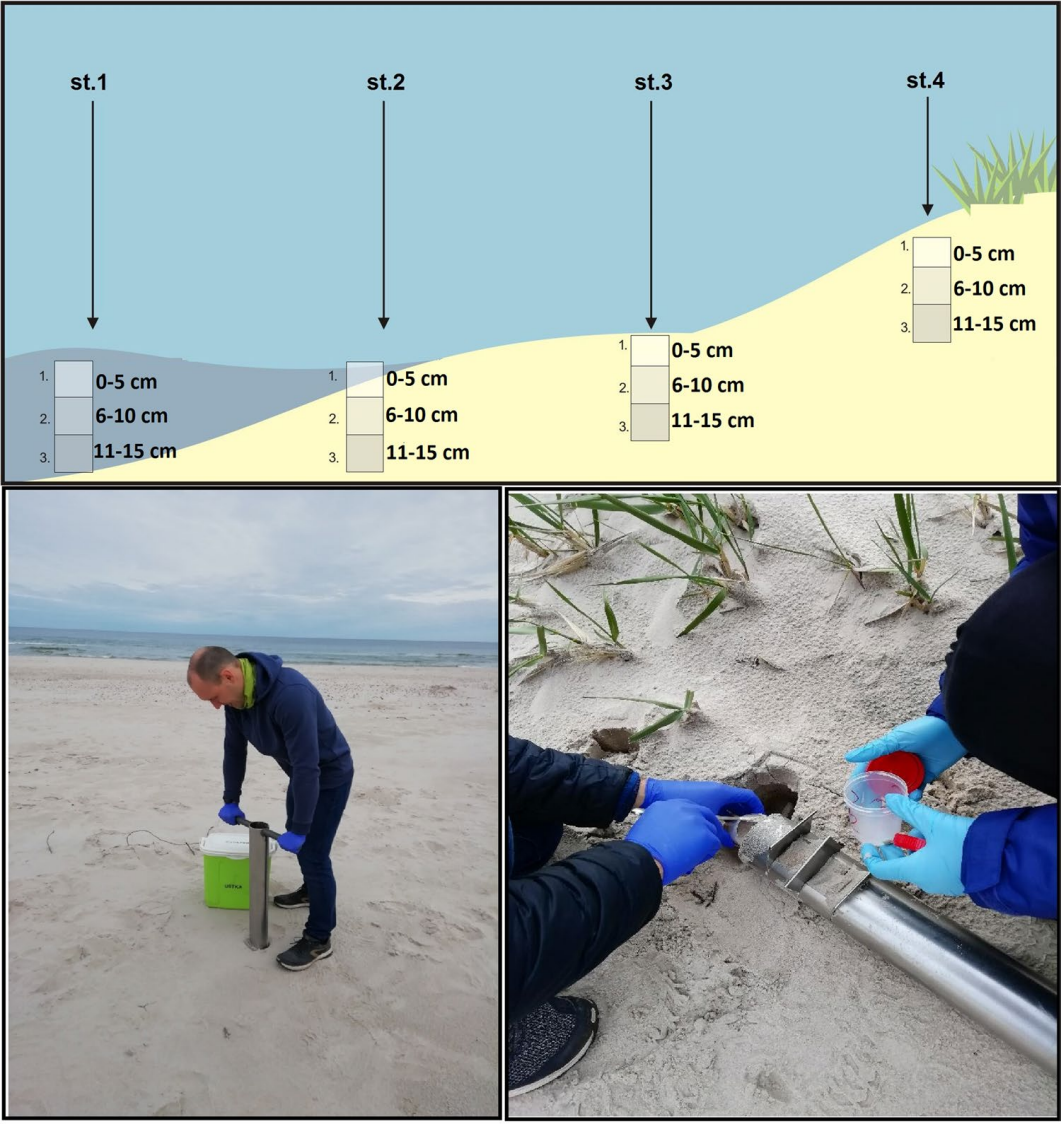
Horizontal and vertical location of the sampling sites. Sand core samples taken with a Morduchaj-Boltowski sampler

Sand samples were taken from four beaches in spring, summer, and autumn in 2017. In each of the studied beaches, a transect was marked along a profile perpendicular to the shoreline, and four sampling sites were located along this transect (Fig. 2):

- Site 1– in the sea, approximately 1–1.5 m from the waterline into the open water at a depth of about 0.5–1 m;
- Site 2 – at the waterline, at the boundary between the beach and the sea;
- Site 3 – halfway up the beach;
- Site 4 – in a sheltered place among dunes.

Sand core samples, three per site, were taken with a Morduchaj-Boltowski sampler (length — 30 cm, inner diameter — 15 cm). Already in the field, 15 cm long sand cores were divided horizontally into three sections: 0—5 cm, 6—10 cm, and 11—15 cm. Sand samples were collected in sterile plastic boxes, which were put into containers with ice, in which temperature did not exceed 4 °C. All samples were transported to the laboratory as soon as possible, and then stored at − 60 °C which ensures high viability of microorganisms [37] for further bacteriological analyses.

### Bacteriological Assay

**Fig. 3:**
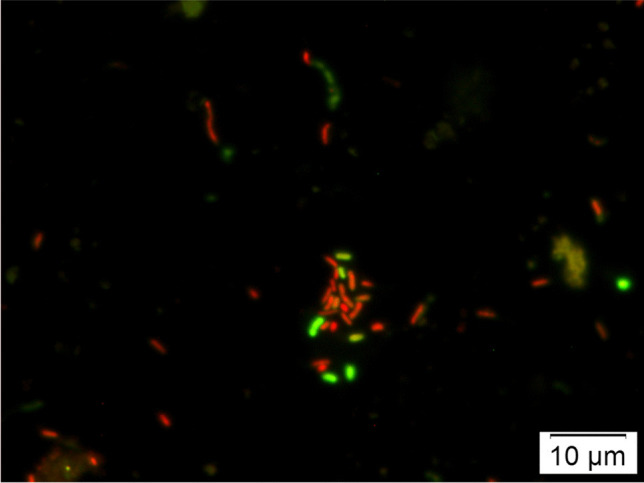
Epifluorescence photomicrography of bacteria inhabited sand of Ustka beach stained by using BacLight viability kit. Dead bacteria show up red with the PI stain and live cells are labelled green with SYTO 9

According to Quéric et al. [38], Ríos et al. [39], and Perliński and Mudryk [29], to estimate the total number of bacteriopsammon (TBN) inhabiting the sand of the studied beaches, and to distinguish between live and dead bacteria, we used the LIVE/DEAD (L/D) BacLight viability kit (L-13152, Molecular Probes, Eugene, OR, USA). In order to determine bacteriological parameters, sand samples were thawed. Then, 5 g of sand was added to 45 ml^−1^ of sterile 8‰ artificial seawater and the sample was sonicated for 60 s in a sonicator (Bandelin Sonoplus HD 2070, 70 W, 20 kHz) in order to desorb bacteria normally stuck to sand grains. A study by Joyce et al. [40] indicated that the short-term sonification of ultrasound used in this study did not significantly affect bacterial cell viability. After sonification, 1 ml of each diluted sample was transferred into capped polyethylene vials and stained according to the instructions attached by the manufacturer. All samples were incubated for 15 min in the dark at room temperature. After incubation, the samples were filtered with a Millipore apparatus through 0.2 μm pore size, 13 mm diameter black, polycarbonate filters. After filtration of the sample, the filter was washed with 1 ml^−1^ of distilled filtered water, dried, and then mounted on microscopic slides. Filters were mounted with low-fluorescent mounting oil (provided with the viability kit) and examined by an epifluorescence microscope (Olympus BX-41) at magnification of × 10 eyepiece and × 100 objective lens, equipped with a filter block B-2A for blue light (EX450-490 excitation filter, DM510 dichroic mirror and BA520 barrier filter; excitation λ = 480/490 nm, emission λ = 500/635 nm) [29] and camera Color Viev. Bacteria were counted in 20 randomly selected fields of view from each layer and sampling site. Green stained cells were classified as “live,” whereas cells stained red were classified as “dead” (Fig. 3). The number of cells in each sample was calculated according to Boulos et al. [41] using the formula T = N × A/a ÷ V where T is the number of bacteria/ml, N is the average number of bacteria/field, A is the surface filtration (mm^2^), a is the area of the microscopic field and V is the volume of a filtered sample (ml). According to Quéric et al. [38], Pearce et al. [28], and Säwström et al. [42], counts of live and dead bacterial cells were summed up to estimate the total bacterial abundance. The number of psammon bacteria was normalized to sand dry weight (d.w.) after drying at 105°Cfor 24 h.

### Statistical Analyses

Statistical parameters (standard deviation — SD, coefficient of variation — CV, coefficient of dispersion — CD) used in the statistical analysis were based on Velji and Albright [43]. The statistical analysis of the obtained studies results was calculated using Statistica 9.0 software. The normal distribution of the data was checked by using the Shapiro–Wilk test before statistical analysis. According to Vignesh et al. [44], if the distribution of the variable met the condition of normality, ANOVA was used to compare the means. When mean values revealed a distribution other than normal, a non–parametric test was used — the Kruskal–Wallis ANOVA rank test and the median test as an equivalent of ANOVA. In order to examine possible inter-relationships between measured bacteriological parameters, discrete, single pair, simple regression analysis was made using Statistica 9.0 software.

## Results

All the results of total psammon bacterial number (TBN) and abundance of live and dead bacterial cells are presented in Table 1. TBN absorbed on the grains of sand from the beaches in Darłowo, Rowy, and Ustka was similar and varied from 5.12 ± 2.25 to 5.65 ± 1.98 × 10^6^ cells × g^−1^ d.w., whereas the lowest (4.10 ± 1.37 × 10^6^ cells × g^−1^ d.w.) TBN was determined in the sand of the beach in Czołpino. Live cells of psammon bacteria constituted 50–53% of bacteria inhabiting the sand of the beaches in Darłowo and Rowy. In Czołpino and Ustka beach sand, a fraction of live bacteria was three times (31–39%) lower than the number of dead bacteria.

**Table 1:** Total bacteria number and enumeration abundance of live and dead cells psammon bacteria number the in sand of studied beaches. Statistical parameters (*SD*, standard deviation; *CV*, coefficient of variation; *CD*, coefficient of dispersion)

Beach bacteria		Statistical parameters					
		Mean (10^6^ cells∙g^−1^ d.w.)	Range (10^6^ cells∙g^−1^ d.w.)	SD	CV [%]	CD	% Live
Czołpino	Live	1.62	0.52–5.84	1.02	63.12	0.65	39.5
	Dead	2.48	0.98–4.67	0.98	39.54	0.39	
	TBN	4.10	2.18–7.62	1.37	33.43	0.46	
Darłowo	Live	2.81	1.41–6.02	1.10	39.37	0.43	50.1
	Dead	2.79	0.66–8.01	1.81	64.76	1.17	
	TBN	5.60	2.77–11.42	2.65	47.25	1.25	
Rowy	Live	2.74	0.36–5.78	1.29	47.03	0.61	53.5
	Dead	2.38	0.61–5.57	1.43	60.27	0.86	
	TBN	5.12	1.84–9.59	2.25	43.90	0.99	
Ustka	Live	1.78	0.65–3.00	0.66	37.31	0.25	31.5
	Dead	3.87	2.08–8.74	1.46	37.64	0.55	
	TBN	5.65	3.08–11.70	1.98	35.09	0.70	

**Fig. 4:**
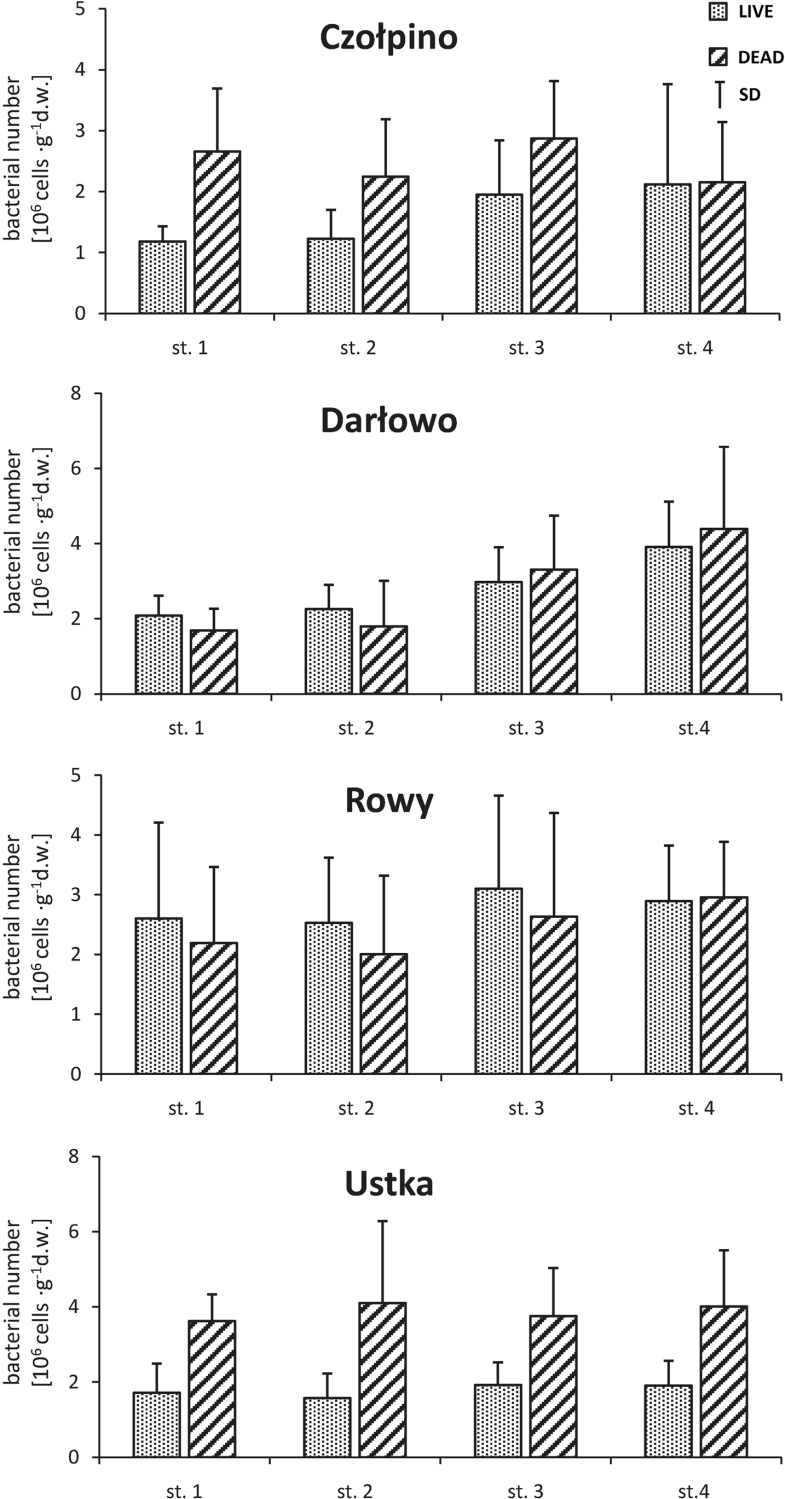
Horizontal variations number live and dead cells bacteria in different parts studied beaches (average from the pooled data of all sand layers and seasons). Vertical bars represent standard errors

The data on the number of live and dead bacterial cells isolated from the transect marked along the profile perpendicular to the shoreline of the studied beaches are given in Fig. 4. The number of live and dead psammon bacteria isolated from the surface of sand grains was different in different parts of the studied beaches. Generally, the highest number of the two studied groups of bacteria inhabiting dry sand of Czołpino, Darłowo, and Rowy beaches was recorded in the middle part of the beach (site 3) and the dune (site 4). In the sand of Ustka beach, the number of active (live) and inactive (dead) psammon bacteria was similar in all horizontal profiles.

**Fig. 5:**
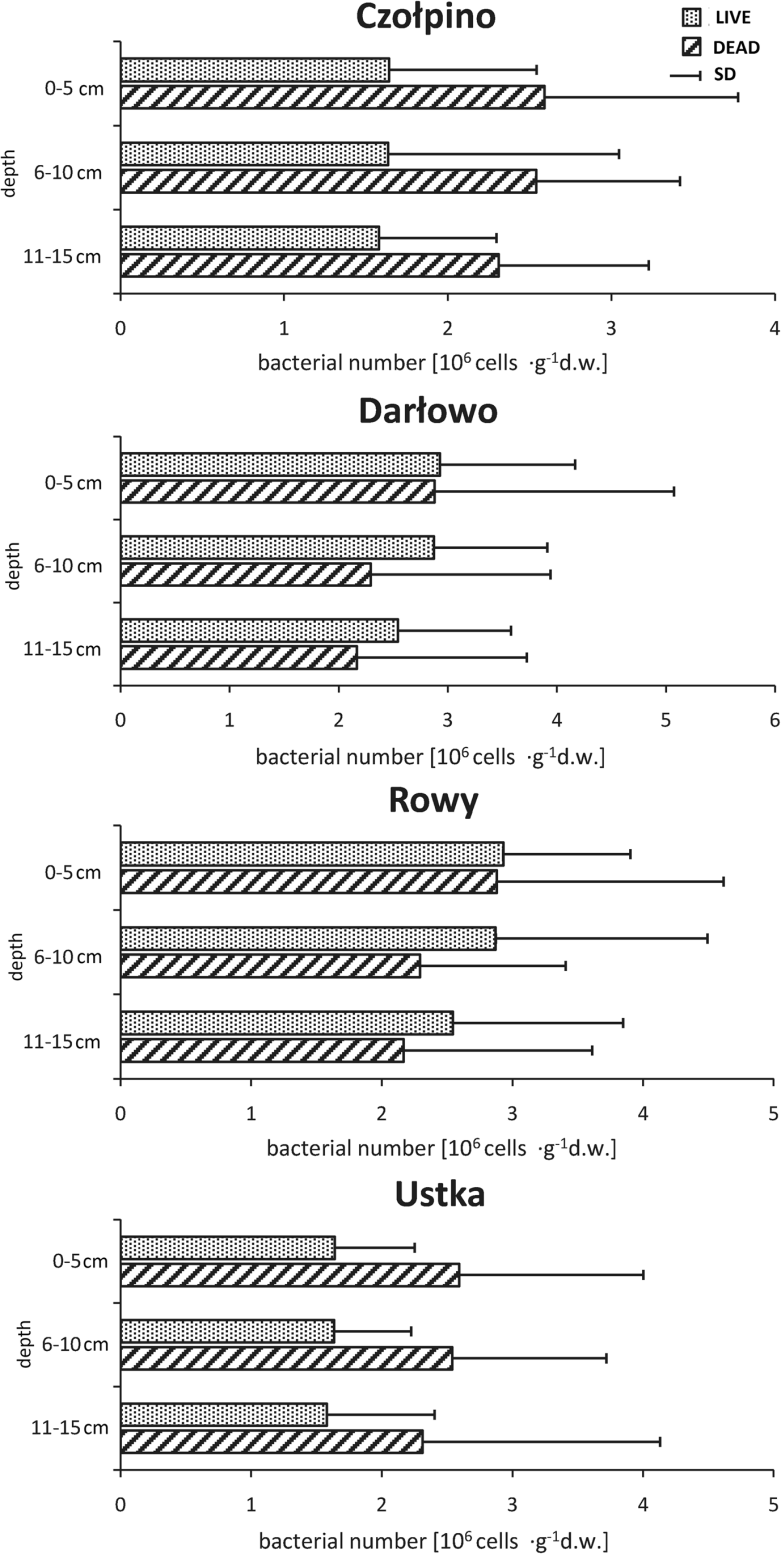
Vertical profiles of the abundance of live and dead bacteria inhabiting sands grains of investigation beaches (average from the pooled data of all sites and seasons). Vertical bars represent standard errors

The data on the number of live and dead psammon bacteria in vertical profiles of the studied beaches are presented in Fig. 5. In the sand of Czołpino, Darłowo, and Rowy beaches, the highest number of two bacterial groups was found in the uppermost (0–10 cm) sand layer and the lowest at the deepest (11–15 cm) sand layer. In the sand of Ustka beach, we noted homogeneous distribution of live and dead bacterial cells in all examined sand cores (0–15 cm).

**Fig. 6:**
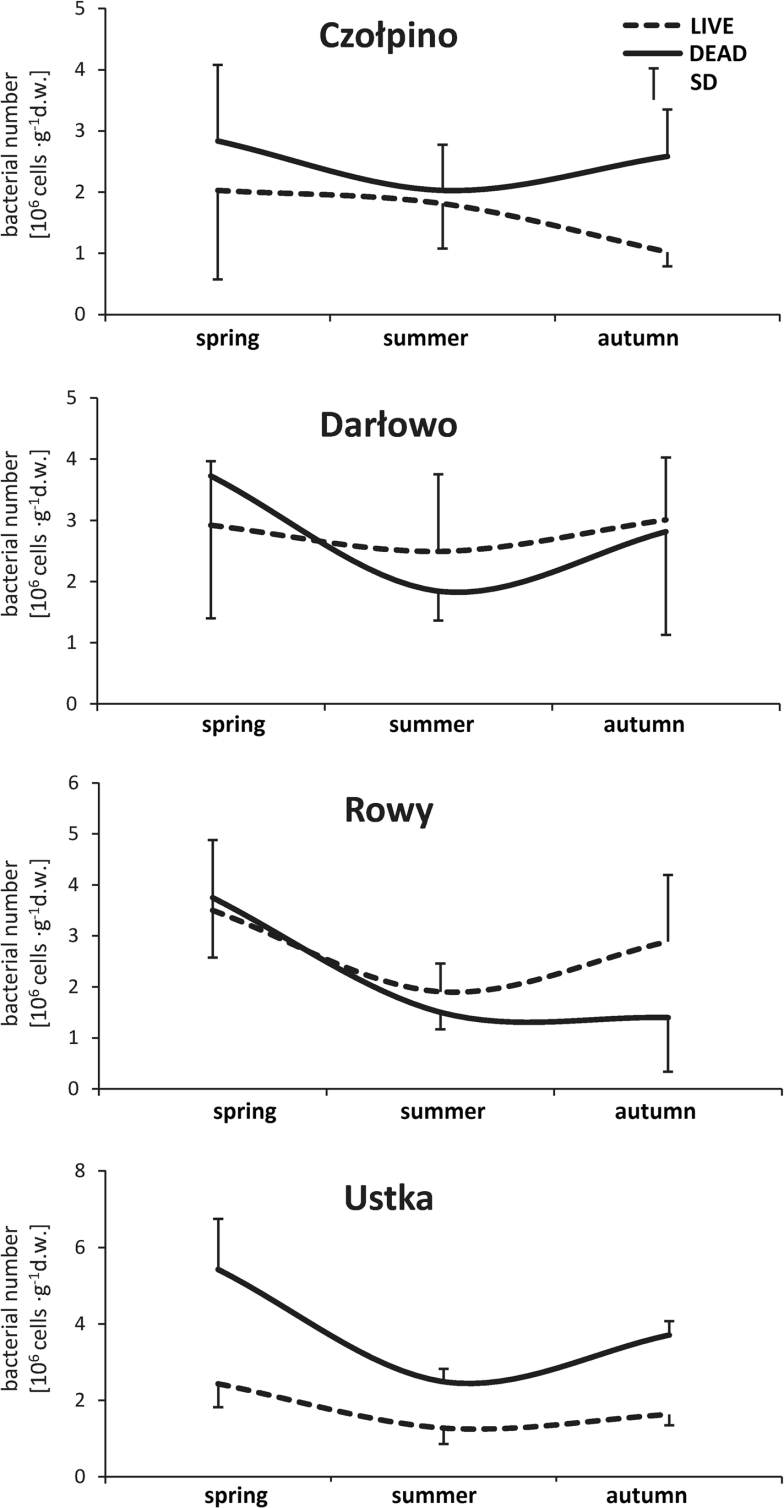
Seasonal dynamics change of number of studied both cells bacteria (average from the pooled data of all sand layers and all sites). Vertical bars indicate standard errors

Based on the data collected in this study (Fig. 6), a distinct seasonal variation in the abundance of live and dead psammon bacteria was shown. Generally, the maximum number of two bacterial groups at four studied beaches was noted in spring. The lower number of active and inactive psammon bacteria was observed in the summer season.

The two-way ANOVA analysis on the beach of Rowy (live — *p* ≤ 0.01 dead — *p* ≤ 0.001) and Kruskal–Wallis test for the beach in Ustka (live — *p* ≤ 0.001 dead — *p* ≤ 0.001) indicated that both number of studied live and dead bacteria displayed significant differences with seasons and on the beach in Darłowo (Kruskal–Wallis test) (live — *p* ≤ 0.01 dead — *p* ≤ 0.001) with sites (Table 2). In the sand of Czołpino, beach number of live bacteria varied significantly with seasons (Kruskal–Wallis test live — *p* ≤ 0.01).

**Table 2:** Analyses of ANOVA of variance and the Kruskal–Wallis and Fisher test due to site, layer and season. Significance (p) level is indicated by asterisks: ** *p* ≤ 0.01, *** *p* ≤ 0.001, *ns*, non-significant level

Beach bacteria		Source of variation	H	F	*p*
Czołpino	Live	Site	5.210		ns
		Layer	0.722		ns
		Season	11.102		**
	Dead	Site		1.085	ns
		Layer		0.267	ns
		Season		2.292	ns
Darłowo	Live	site	14.055		**
		Layer	1.423		ns
		Season	2.572		ns
	Dead	Site	17.206		***
		Layer	1.626		ns
		Season	3.302		ns
Rowy	Live	Site		0.387	ns
		Layer		0.111	ns
		Season		7.964	**
	Dead	Site	1.136		ns
		Layer	0.706		ns
		Season	24.734		***
Ustka	Live	Site	1.951		ns
		Layer	0.126		ns
		Season	16.803		***
	Dead	Site	0.385		ns
		Layer	0.092		ns
		Season	28.578		***

Explanations: *F*, Fisher test in ANOVA of variance; *H*, the Kruskal–Wallis test; *p*, significance level; *ns*, non-significant

**Fig. 7:**
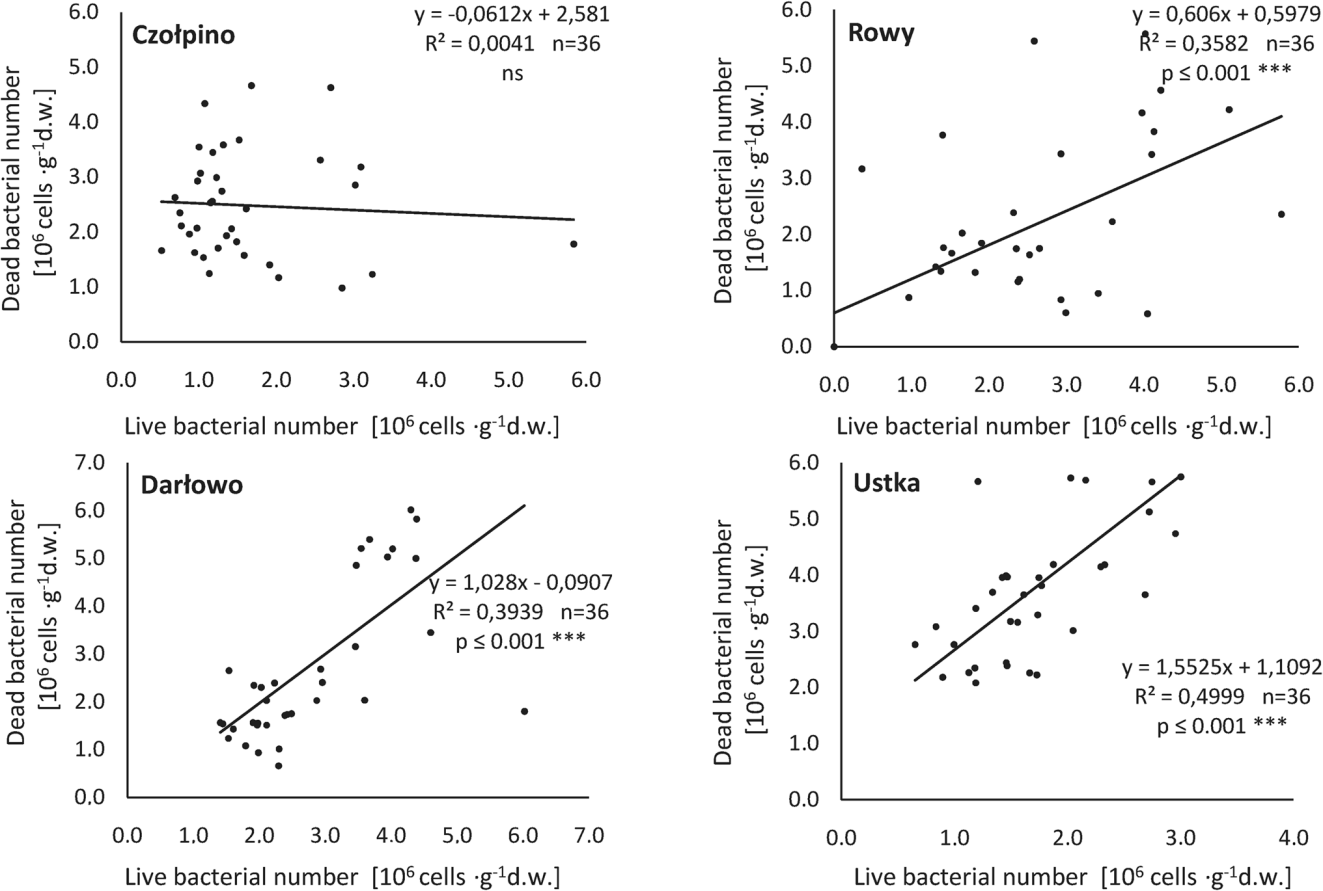
Relationships between the abundance of live and dead bacteria Solid line represents linear regression including all data (y — regression equation, *R*.^2^ — coefficient of determination, n — number of samples, p — significance level)

Statistical analysis of the examined bacteriological parameters, which was based on linear regression (Fig. 7) showed that there was a highly (*p* ≤ 0.001) significant positive correlations between number of live and dead bacteria inhabited sand grains in the Darłowo (*R*^2^ = 0.39), Rowy (*R*^2^ = 0.35) and Ustka (*R*^2^ = 0.49) beaches. In the sand of Czołpino beach, no statistical correlation was found between the number of both the studied bacterial groups.

## Discussion

Bacteria are now recognized as a major biological force decisive for functioning of marine ecosystems, for example marine beaches [5, 45]. Knowledge of the number of bacteria and their metabolic activity is very important in the studies of microbial secondary production, bacterial growth rates, and division [13, 29, 46]. The total bacterial number in the studied sandy beaches varied from 4.1 to 5.6 × 10^6^ cells × g^−1^ d.w. This range was consistent with those reported from other beaches, such as sandy beaches located in Puck (Poland) (2.9–16.1 × 10^6^ cells × g^−1^ d.w.) [3], Collelungo beach (Italy) (2.0–15.0 × 10^6^ cells × g^−1^ d.w.) [47], Nova Scotia (Canada) (19.0–73.0 × 10^6^ cells × g^−1^ d.w.) [48] and Sopot (Poland) (34.0–91.0 × 10^6^ cells × g^–1^ d.w.) [49] but lower (0.9–1.8 × 10^8^ cells × g^−1^ d.w.) than the values recorded on Baia Blu beach (Italy) [50]. The authors of this study are fully aware that the determined number of microorganisms may be underestimated during sonification due to insufficient desorption from the sand grains; however, studies by Lee et al. [51] on soil samples showed that sonification lasting up to 3 min gives the highest results in desorbing bacterial cells from sediment particles.

Despite the ecological role of benthic bacteria in biogeochemical cycles at a global scale, information on the fraction of live versus dead bacterial cells in marine sediments is very important in ecological studies [24, 52, 53]. Natural bacterial assemblages display different metabolic levels and vital states and only a part of aquatic bacteria are live and actively growing, while a large fraction is dormant and dead [24, 26, 53]. The results of our study also showed that among all bacteria recorded on the surface of sand grains on the four studied beaches, only 31 to 53% were live cells. Sherr et al. [54] and Pearce et al. [28] suggest that only a small fraction of bacterial community in the studied marine beaches was metabolically active and contributed to bacterial secondary production, growth and division, respiration and enzymatic activity in that marine environment. These results are in accordance with previous studies conducted in lakes [42], rivers [55], ponds [26], estuaries [29], coastal [56] and marine ecosystems [24, 53, 57]. According to Davidson et al. [26] and Papageorgiou et al. [58], a low abundance of live bacterial cells may result from grazing these metabolically active organisms by protozoa especially bacterivorous nanoflagellates and also meio- and macrofauna. Numerous authors [59, 60] reported size-specific grazing of bacterivores leading to preferential grazing of live, active and/or dividing bacteria. Grazing selectivity may result in active bacteria being grazed at rates around 4 times that of inactive cells [26, 61]. This process has been identified as a dominant factor modifying and controlling bacterial number and mortality in aquatic ecosystems [60]. Another factor that influences the abundance of live bacterial cells are viruses [46]. According to Maranger et al. [62], viral infections of active bacteria may be responsible for a large fraction of live bacteria mortality in water basins. Viral infection is known to impair the integrity of bacterial cell walls [42, 63]. It has been suggested that viruses may cause up to 30% mortality of bacteria in aquatic ecosystems [42, 64].

The interactions between sand and sea on marine beach transfer bacteria between different parts of this ecotone [7, 12]. For this reason, horizontal zonation of the distribution of psammon bacterial communities is a well-known global phenomenon on marine sandy beaches [19, 65]. The results of the present study also support these findings. In our study especially on the beach in Ustka, some difference in the abundance of live and dead psammon bacteria along the horizontal profile was observed. However, in the case of the beach in Darłowo, a clear trend of increase in the abundance of both studied groups of bacteria with distance from the shoreline was noted. On the studied beaches, the maximum density of bacteria was noted in the dune and the middle part of the beach (dry zones), whereas bacteria were much less abundant in wet zones, i.e., in the sand samples collected under seawater surface and at the swash zone. According to Schumann et al. [46] and Podgórska and Mudryk [19], a high number of bacteria recorded in the dune and middle part of the beach may result from the accumulation of considerable amounts of particular and dissolved organic matter of anthropogenic, plant and animal origin. These organic compounds can generate growth, production, and high number of bacteria in these zones of marine beach. Moreover, dune which acts as a biocatalytic filter is enriched with humic substances originating from decaying roots of grasses growing on it [66, 67]. Humic substances may be utilized by bacteria very intensively in metabolic processes as a good source of carbon or energy [68]. The low number of bacteria in the sand at waterline as well as under seawater surface may be due to grazing pressure of bacterivorous organisms, which, as has been shown by Haque et al. [69], are very numerous in these parts of the beach. Nanoflagellates, ciliates, macrofauna, and meiofauna for which bacteria represent a readily available source of food and main energy input, are important factors controlling the numbers of bacteria in wet sand on marine beaches [2, 47, 58]. The low number of bacteria in wet sand may be also due to a high-energy character of this marine beach region which is an inhospitable place for bacteria not able to cope with sheer stress associated with a high energy environment [48, 58].

In the sand of Czołpino, Darłowo, and Rowy beaches, the highest number of two studied bacterial groups was found in the surface sand layer and it decreased with increasing sediment depth. Previous studies carried out on marine sediments [19, 65, 70] demonstrated the characteristic vertical distribution of bacteria. These organisms were the most abundant in the top layer of sand, while their number significantly decreased with increasing bottom depth. The concurrent decline in bacterial number with increasing sediment depth might be determined by vertically changing different environmental conditions [38, 71]. This characteristic vertical distribution of psammon bacteria in sand of marine beaches most probably results from the fact that temperature [20], better mixing of sand (also caused by tourist movement) [3], concentration of organic matter [34] as well as better oxygenation [72], which are main stimulators of growth for psammon bacteria, decrease with depth. In the sand of Ustka beach, the fraction of live and dead bacteria was not significantly different between all examined depths. This homogenous distribution of psammon bacteria in the vertical profile on this beach could have been the result of annual dredging, which supplies this beach with huge amounts of sediments collected from the seabed of the Baltic Sea. This process may disturb typical heterogeneous vertical structure of this beach.

The results of our study showed seasonal dynamics in the number of live and dead bacteria inhabiting sand of all studied beaches. Generally the maximum number of two bacterial groups was recorded in spring and their minimum was noted in summer. This finding supports earlier studies carried out in seawater and coastal water in the Netherlands [57], in the Warnow River in the northeast Germany [55], and the Słupia River estuary in Poland [29]. According to Davidson et al. [26], Alonso-Saez et al. [73], and Freese et al. [55], lower number of live and dead psammon bacteria in summer in beaches may result from bacteriostatic or bactericidal inhibitory effect on these organisms due to intensified sunlight insolation, especially with the shortest wavelength fraction of ultraviolet radiation. Solar ultraviolet radiation (290 to 400 nm) causes cellular damage on different cell targets, including nucleic acids, proteins and lipids, which may end up in mutations, cell inactivation, and death [74, 75]. Besides the negative influence of solar radiation, Perliński and Mudryk [29] indicated that excretion of bacteriostatic and bactericidal substances, particularly those synthesized by cyanobacteria and chlorophyta during summer algal bloom in seawater flooding, the sandy beach swash zone may be another factor causing increased psammon bacteria mortality at this time of the year.

## Conclusion

The psammon bacteria play a central role in regulating accumulation, export, and transformation of the largest part of organic matter in the marine ecosystems, for example marine beaches. Therefore, the number of bacteria and their metabolic activity influence microbial secondary production, bacterial growth rates and division. Among all bacteria recorded on the surface of sand grains on four studied beaches, only 31 to 53% were live cells, metabolically active, and they are important in microbial loop, biological balance, and for functioning of these marine beach ecosystems. In the sand of Darłowo beach, a clear difference in the abundance of live and dead psammon bacteria was observed along the horizontal profile. This is the example of well-known bacteria transfer between sand and sea in marine beach ecotone. Figure 5 shows the results of the present study in the sand of Czołpino, Darłowo, and Rowy beaches demonstrated the characteristic (but not statistically significant) vertical distribution of bacteria. The psammon bacteria were the most abundant in the top layer of sand, while their number significantly decreased with increasing bottom depth. Seasonal dynamics in the number of live and dead bacteria inhabiting sand of all studied beaches was observed too. Generally, the maximum number of two bacterial groups was recorded in spring and their minimum was noted in summer. Many physical, chemical, and biological factors may be causing changes in the number of psammon bacteria at different time of year.

## Data Availability

Data will be made available on reasonable request.
